# The Antimicrobial Peptide Histatin-5 Causes a Spatially Restricted Disruption on the *Candida albicans* Surface, Allowing Rapid Entry of the Peptide into the Cytoplasm

**DOI:** 10.1371/journal.ppat.1000190

**Published:** 2008-10-31

**Authors:** A. Brian Mochon, Haoping Liu

**Affiliations:** Department of Biological Chemistry, University of California, Irvine, California, United States of America; Carnegie Mellon University, United States of America

## Abstract

Antimicrobial peptides play an important role in host defense against microbial pathogens. Their high cationic charge and strong amphipathic structure allow them to bind to the anionic microbial cell membrane and disrupt the membrane bilayer by forming pores or channels. In contrast to the classical pore-forming peptides, studies on histatin-5 (Hst-5) have suggested that the peptide is transported into the cytoplasm of *Candida albicans* in a non-lytic manner, and cytoplasmic Hst-5 exerts its candicidal activities on various intracellular targets, consistent with its weak amphipathic structure. To understand how Hst-5 is internalized, we investigated the localization of FITC-conjugated Hst-5. We find that Hst-5 is internalized into the vacuole through receptor-mediated endocytosis at low extracellular Hst-5 concentrations, whereas under higher physiological concentrations, Hst-5 is translocated into the cytoplasm through a mechanism that requires a high cationic charge on Hst-5. At intermediate concentrations, two cell populations with distinct Hst-5 localizations were observed. By cell sorting, we show that cells with vacuolar localization of Hst-5 survived, while none of the cells with cytoplasmic Hst-5 formed colonies. Surprisingly, extracellular Hst-5, upon cell surface binding, induces a perturbation on the cell surface, as visualized by an immediate and rapid internalization of Hst-5 and propidium iodide or rhodamine B into the cytoplasm from the site using time-lapse microscopy, and a concurrent rapid expansion of the vacuole. Thus, the formation of a spatially restricted site in the plasma membrane causes the initial injury to *C. albicans* and offers a mechanism for its internalization into the cytoplasm. Our study suggests that, unlike classical channel-forming antimicrobial peptides, action of Hst-5 requires an energized membrane and causes localized disruptions on the plasma membrane of the yeast. This mechanism of cell membrane disruption may provide species-specific killing with minimal damage to microflora and the host and may be used by many other antimicrobial peptides.

## Author Summary

In most healthy individuals, the yeast *Candida albicans* is found within the oral cavity as part of the normal microflora. Though under immunocompromising conditions, this benign microbe can become an opportunistic pathogen causing oral candidiasis (i.e. thrush). Although antifungal drugs are typically efficacious, the paucity of drugs and their increasing usage has led to rising drug resistance. Thus, researchers have begun to look at alternative therapeutics, such as the candidacidal salivary peptide histatin-5. To date, little is known about the initial binding and subsequent internalization that facilitates Hst-5's killing activity. Thus, our study attempted to determine how Hst-5 is internalized into *C. albicans*. It was thought that Hst-5 is transported into the cytoplasm without disruption of the plasma membrane. However, our study finds that Hst-5, under physiological concentrations, disrupts the plasma membrane and is rapidly translocated into the cytoplasm, leading to cell death. Interestingly, the internalization of Hst-5 is initiated from a single spatially restricted site on the plasma membrane rather than multiple breaches on the cell surface. This novel mechanism of membrane disruption provides new insights into how Hst-5 and other antimicrobial peptides may be acting on pathogenic microorganisms.

## Introduction

Oral candidiasis is most commonly associated with individuals infected with the human immunodeficiency virus (HIV), and it is also seen in infants, patients with diabetes mellitus, and those receiving broad-spectrum antibiotics. However, oral candidiasis is relatively uncommon in the general population, despite the fact that *Candida albicans* can be recovered in the alimentary canal of healthy individuals in over 50% of cases [1]. Antimicrobial peptides, including histatins, play an important role in the innate defense against oral *Candida* infections.

Histatins are a family of low-molecular weight histidine-rich cationic peptides that are found specifically in human salivary secretions [2]. Histatin-5 (Hst-5), a 24-residue peptide is the most potent member of the family with respect to fungicidal activity against *C. albicans*. It kills *C. albicans in vitro* at physiological concentrations (15–30 µM) [3],[4]. Structural studies of Hst-5 have shown that it takes on a random coil structure in aqueous solvents and adopts a largely α-helical conformation in non-aqueous solutions [5],[6]. Many antimicrobial cationic peptides form either α-helical or β-sheet structures that exhibit a strong amphipathic nature. After the initial electrostatic attraction to an anionic microbial surface, these cationic amphipathic peptides can spontaneously insert into cell membranes and form pores/channels, causing lysis of the cell membrane [7],[8]. However, unlike the classical pore-forming peptides, Hst-5 is predicted to lack sufficient amphipathic character to insert spontaneously into microbial membranes [3],[6]. Consistent with this prediction, Hst-5 has little lytic effect in a liposome model system [9].

The activity of Hst-5 against *C. albicans* is believed to be initiated through cell wall binding, followed by translocation and intracellular targeting. Hst-5 has been shown to localize to the cytoplasm of *C. albicans* cells, where it associates with the energized mitochondria and inhibits respiration [10],[11]. Cytoplasmic Hst-5 also affects cell membrane functions. Hst-5 induces a sizeable noncytolytic efflux of ATP and potassium and magnesium ions into the extracellular milieu, causing a loss in cell volume and ionic imbalance to the yeast cell [12]–[15]. On the other hand, Hst-5 has also been reported to cause slow depolarization of the cytoplasmic and mitochondrial membranes, indicating a lytic activity towards the membranes [16]. Cells treated with Hst-5 also have elevated cell permeability to the small cationic dye propidium iodide (PI) as shown by FACS analysis [10]. Internalization of Hst-5 by *C. albicans* cells is tightly linked to killing and is dependent on cellular metabolism [16]–[18]. Oxygen depletion or inhibition of respiration by sodium azide blocks the translocation of Hst-5 into the cell by rigidifying the cell membrane [10],[17]. The initial binding of Hst-5 to the *C. albicans* cell surface is thought to be initiated by specific binding to Ssa1/2, a heat shock protein present on the cell surface of the yeast [19],[20]. Cells without Ssa2 show an impaired Hst-5 uptake [20]. However, how Hst-5 is internalized is not known. The initial cell surface binding by Hst-5 does not lead to cellular lysis as seen with other antimicrobial peptides [12].

Most polar macromolecules are excluded from the interior of cells due to the impermeability of the plasma membrane. The typical mechanism of entry of extracellular components into yeast cells is dependent on either fluid-phase or receptor-mediated endocytosis [21]–[23]. Recently, cationic cell-penetrating peptides, such as HIV Tat and polyarginine, have been shown to translocate directly from the extracellular surface into the cytoplasm without the need for endocytic vesicles in both yeast and mammalian cells [24],[25]. This novel internalization requires cationic charge and an electrostatic interaction with cell the surface.

This study aimed to determine how Hst-5 is translocated into the cytoplasm of *C. albicans*. To date, the mode of internalization of Hst-5 is not defined, though it has been suggested that internalization of the antifungal peptide is initiated through a non-lytic manner by an unidentified translocase [26]. Here we report that the internalization event is actually a non-classical lytic event. We visualized an immediate and rapid internalization of Hst-5 and fluorochromatic dyes into the cytoplasm from a spatially restricted site on the plasma membrane, and a concurrent rapid expansion of the vacuole. Cell death is completely correlated with the appearance of cytoplasmic Hst-5. Our study provides the first direct evidence for a breach in the plasma membrane as the initial damage by extracelluar Hst-5 on *C. albicans* and a mechanism of its internalization into the cytoplasm.

## Results

### The intracellular localization of histatin-5 is concentration-dependent

**Figure 1:**
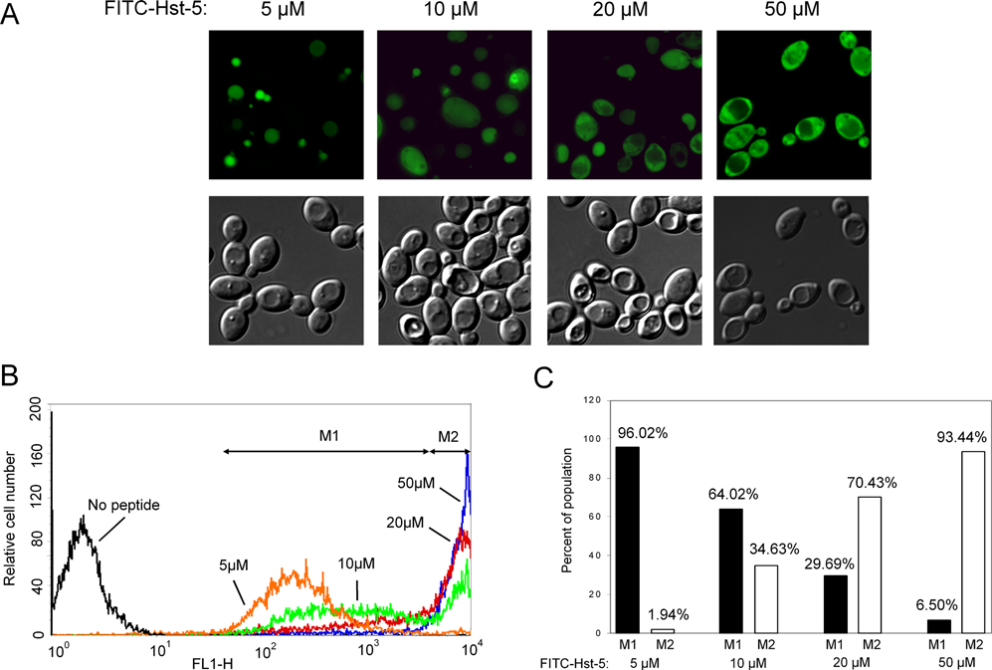
Concentration-dependent localizations of FITC-Hst-5. *C. albicans* cells were incubated for 30 minutes with 5, 10, 20, and 50 µM FITC-Hst-5. (A) Fluorescent and DIC images of cells with FITC-Hst-5 at the indicated concentrations and (B) flow cytometry analysis of the same cells. The concentration dependent localization is represented by the markers M1 (vacuole) and M2 (cytoplasm). (C) The bar graph represents the percentage of the population where Hst-5 is localized to the vacuole (M1) and to the cytoplasm (M2) for the varying concentrations.

Hst-5 at physiological concentration (10–30 µM) has been found distributed throughout the cytoplasm of *C. albicans* cells [27],[28]. However, by using FITC-conjugated Hst-5 at 10 µM or 20 µM, we observed two populations of cells with distinct Hst-5 localization (Figure 1A). In some cells, Hst-5 was localized strictly to the vacuole, but not in the cytoplasm; in other cells, Hst-5 was localized throughout the cytoplasm. The internalization of the FITC-conjugated Hst-5 produced varying fluorescent intensity in both the vacuolar- and cytoplasmic-associated cells. It seemed that more cells showed vacuolar localization at 10 µM than at 20 µM Hst-5, and the reverse was seen for cells with cytoplasmic localization at these concentrations. To examine a possible effect of Hst-5 concentration on its cellular localization, we examined cells in 5 µM and 50 µM Hst-5. Strikingly, cells exposed to 5 µM Hst-5 showed mostly vacuolar localization and cells in 50 µM showed largely cytoplasmic localization with little vacuolar fluorescence (Figure 1A). Therefore, with increasing Hst-5 concentrations, we observed a shift in cell population from vacuolar to cytoplasmic localization. Because the fluorescent intensity of cells with cytoplasmic Hst-5 was much higher than that of cells with vacuolar Hst-5, we were able to quantify the percentage of cells in each population by flow cytometry (Figure 1B and 1C). Cells with vacuolar Hst-5 are in the peak with low intensity on the left, and cells with cytoplamic Hst-5 are in the high intensity peak on the right. Moreover, with increasing Hst-5 concentrations, more cells are shifted from the low intensity peak to the high intensity peak in flow cytometry (Figure 1B and 1C). Since the majority of cells with cytoplasmic Hst-5 still had enlarged intact vacuoles that excluded Hst-5, the concentration dependent localization of Hst-5 is likely through two distinct pathways of internalization.

### Vacuolar uptake of histatin-5 is dependent on receptor-mediated endocytosis

**Figure 2:**
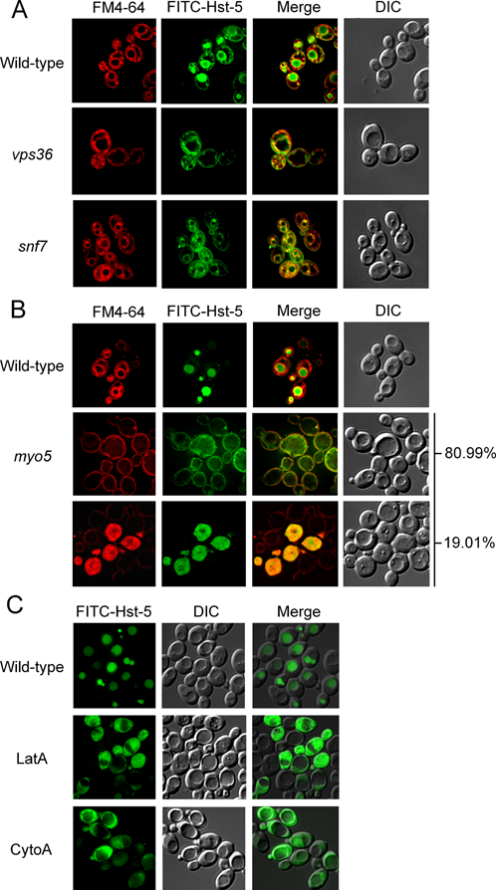
Vacuolar localization of FITC-Hst-5 is dependent on receptor-mediated endocytic pathway. (A) *C. albicans* wild-type, *vps36*, and *snf7* cells were incubated for 30 minutes with 10 µM FITC-Hst-5 and 10 µM FM4-64 at 30°C. (B) *C. albicans* wild-type and *myo5* cells were incubated for 10 minutes with 10 µM of FITC-Hst-5 and 10 µM FM4-64 at 30°C. The populations of plasma membrane and cytoplasmic localization, 80.99% and 19.01% as indicated, were determined by flow cytometry. (C) *C. albicans* wild-type cells were treated with either 50 µM latrunculin A or 5 µM cytochalasin A for 60 minutes at room temperature. The cells were then exposed to 5 µM FITC-Hst-5 for 30 minutes at 30°C.

The vacuolar uptake of Hst-5 could be through either receptor-mediated or fluid-phase endocytosis. To determine which is responsible for vacuolar Hst-5 uptake, mutants that block receptor-mediated, but not fluid-phase, transport to the vacuole were used. Monoubiquitination of transmembrane receptors at their cytoplasmic domains is required for the sorting of these integral membrane proteins into the luminal vesicles of multivesicular bodies (MVBs). ESCRT (endosomal sorting complex) complexes are essential for the sorting event. Receptors that normally internalize and traffic into MVBs and subsequently degrade in the vacuole are instead accumulated on the membrane of MVBs in the mutants of *VPS36* or *SNF7*, components in the ESCRT complexes [23], [29]–[31]. After incubating *C. albicans* wild-type, *vps36*, and *snf7* mutant cells in 10 µM Hst-5 and 10 µM FM4-64, a lipophilic membrane dye, for 30 minutes at 30°C, Hst-5 was found to accumulate on the surface of, but excluded from the lumen of vacuole-like membrane structures in *vps36* and *snf7* mutant cells (Figure 2A). It should be noted that FM4-64 increased the necessary threshold concentration of Hst-5 in order to observe vacuolar or cytoplasmic-associated Hst-5 localization. These results indicate that Hst-5 is internalized to the vacuole by a receptor-mediated endocytic pathway.

Next, we evaluated the effect of blocking the initial internalization step of endocytosis on Hst-5 uptake. The actin cytoskeleton and type I myosins are critical for the initial invagination event at the cell surface [21],[32]. Mutation of the type I myosin gene (*MYO5*) in *C. albicans* is also deficient in endocytosis, as the uptake of FM4-64 is impeded at the plasma membrane [33]. When *myo5* mutant cells were incubated with 10 µM Hst-5 and 10 µM FM4-64 for 10 minutes at 30°C, both FM4-64 and Hst-5 were found on the plasma membrane, but not in the vacuole in the majority of the cell population (Figure 2B). This observation is consistent with the reported defect of *myo5* cells in endocytosis. Surprisingly, a moderate proportion of the population, 19.01%, had Hst-5 localized within the cytoplasm. Similarly, in cells treated with latrunculin A (LatA) or cytochalasin A (CytoA), which prevents actin polymerization, Hst-5 at 5 µM was observed in the cytoplasm in a majority of the cells, especially CytoA-treated cells, but was not observed in the vacuole or on the plasma membrane (Figure 2C). Therefore, cells without functional actin cytoskeleton greatly decreased the threshold necessary to induce cytoplasmic Hst-5 translocation.

### The cationic charge of histatin-5 is critical for its translocation into the cytoplasm

**Figure 3:**
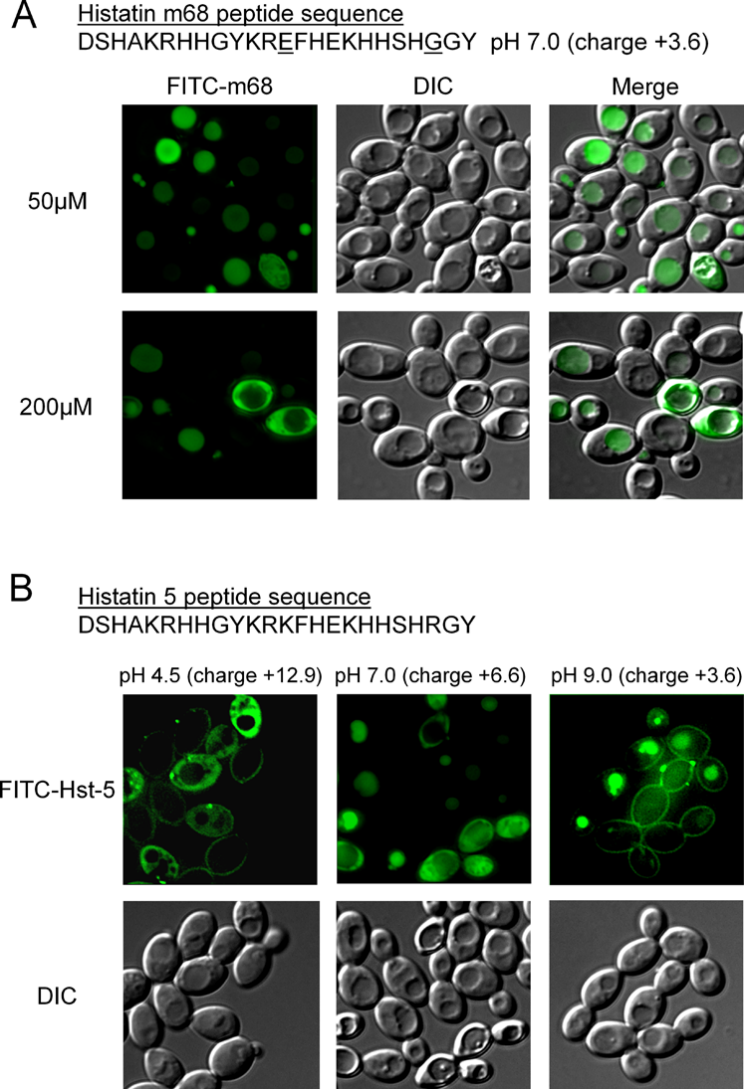
The cationic charge of Hst-5 is important for its uptake into the cytoplasm. (A) *C. albicans* cells were incubated with 50 µM and 200 µM FITC-Hst-5 m68 for 30 minutes at 30°C. (B) *C. albicans* yeast cells were treated with 20 µM FITC-Hst-5 for 30 minutes at 30°C under different pH conditions (4.5, 7.0, and 9.5) as indicated.

The cationic charge of antimicrobial peptides is critical for the initial electrostatic attraction of the peptides to negatively charged cell membranes, and there is a strong correlation between peptide cationicity and antimicrobial activity [34]. For Hst-5, cationic charge is also important for its candicidal activity. A single lysine substitution for a histidine or amidation of the C-terminus of Hst-5 analogs increases the candidacidal activity of the peptide by almost two-fold [35],[36]. Whereas, replacement of lysine-13 with glutamic acid and arginine 22 with glycine, a variant of Hst-5 (m68) reported by Tsai et al., caused a significant reduction in its killing ability [37]. The amino acid substitutions in m68 reduce the cationic charge at pH 7.0 from 6.6 in Hst-5 to 3.6. To determine whether the cationic charge of Hst-5 affects how the antimicrobial peptide is internalized into the fungal cell, we observed the localization of a FITC-conjugated m68 in *C. albicans*. When cells were exposed to concentrations of 5, 10, 20, 50, and 200 µM FITC-m68 at 30°C for 30 minutes, cytoplasmic m68 was not observed at 5, 10, and 20 µM (data not shown) and was only seen in a small portion of the cell population at 50 and 200 µM m68 (Figure 3A). In contrast to Hst-5 at these concentrations, FITC-m68 was localized to the vacuole in the majority of cells (Figure 3A). The result suggests that a robust cationic charge is necessary for cytoplasmic localization of Hst-5.

Since Hst-5 sequence is 29% histidine, which has a pI at around physiological pH, the peptide is expected to have different charges at pHs 4.5, 7.0, and 9.0, which are within the resting pH range of the oral cavity [38]. By incubating *C. albicans* with 20 µM Hst-5 in varying pHs, we could examine the effect of cationic charge on the peptides cellular localization. In cells exposed to Hst-5 at pH 9.0, giving the peptide a charge of +3.6, the majority of the cells showed either cell surface or vacuolar localization, while no cytoplasmic localization was observed. Conversely, when the cells were incubated with Hst-5 at pH 4.5, which induces a positive net charge of 12.9, a preponderance of cells had Hst-5 localized within the cytoplasm and only a small population had the antimicrobial peptide bound to the cell surface (Figure 3B). This is consistent with the previous observation that a strong cationic charge is important for the cytoplasmic localization of Hst-5. However, extreme pHs may have effects on cell physiology that potentially could influence the uptake of Hst-5. Indeed, endocytosis has been shown to be impaired at a low pH [39], which may account for why vacuolar Hst-5 was not observed at pH 4.5. Therefore, Hst-5 uptake pathway is likely affected by both the pH-dependent cationic charge of Hst-5 and pH effects on cell physiology. Nonetheless, we have shown through both amino acid substitution and the ionization of histidine using the pH range of the oral cavity that a strong cationic charge determines the mode of uptake for Hst-5.

### Cytoplasmic localization of histatin-5 is linked to killing, whereas vacuolar histatin-5 is non-cytotoxic

**Figure 4:**
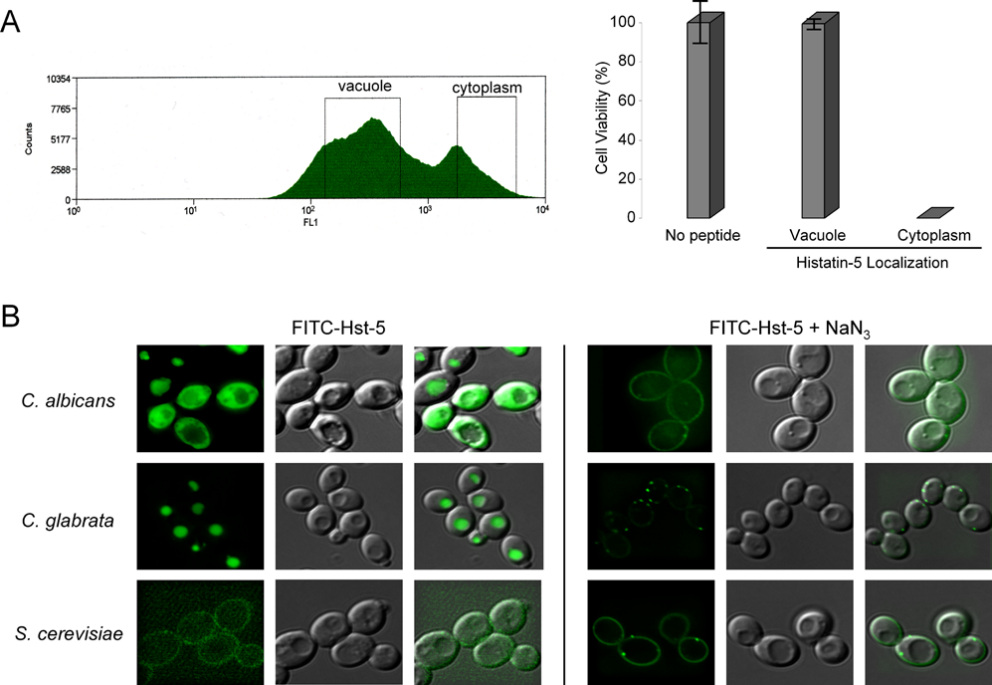
Cytoplasmic localization of Hst-5 is linked to its killing activity. (A) *C. albicans* cells were incubated for 30 minutes with 10 µM FITC-Hst-5 at 30°C. The cells were sorted by gating the two peaks of the histogram representing vacuolar and cytoplasmic localization. The sorted cells were then plated onto YPD plates and incubated overnight at 30°C (data as a mean±1SD of triplicate cultures). (B) *C. albicans*, *C. glabrata*, and *S. cerevisiae* were incubated with either 50 µM FITC-Hst-5 or 50 µM FITC-Hst-5 and 10 mM NaN_3_ for 30 minutes at 30°C. The cells treated with and without sodium azide had equal fluorescence exposure times of 900 and 9 milliseconds, respectively.

Hst-5 at 10 µM kills with around 50% efficiency in a population of 10^6^ cells/ml. Since this concentration gives a mixed population of cells with either vacuolar or cytoplasmic Hst-5, we were interested in determining whether cells with different Hst-5 localizations had different fates. We have observed that cells with cytoplasmic Hst-5 have higher fluorescence intensity than cells with vacuolar Hst-5, and the difference in fluorescence intensity is sufficient to allow us to separate cells into two populations with different Hst-5 localizations (Figure 1). After incubating *C. albicans* cells in 10 µM Hst-5 at 30°C for 30 minutes, cell sorting was carried out to give us two distinct populations of Hst-5 localized cells (Figure 4A), which were confirmed by fluorescence microscopy. Approximately two hundred cells from each population were aliquotted onto plates to determine colony-forming units. Cells from a no-peptide control and cells with vacuolar Hst-5 showed 100% survival. Conversely, none of the cytoplasmic-localized cells produced colonies (Figure 4A). These results provide irrefutable evidence that cytoplasmic Hst-5 is linked to killing, whereas vacuolar Hst-5 is non-cytotoxic to the cells and is maintained in the vacuole.

### Resistance of *C. glabrata* and *S. cerevisiae* to the peptide correlates with a lack of histatin-5 translocation into the cytoplasm

Previous research has shown that the yeasts *C. glabrata* and *S. cerevisiae* show a marked resistance to Hst-5 [40]–[42]. We wanted to determine if their insensitivity to the antimicrobial peptide was due to the lack of cytoplasmic translocation of Hst-5. The yeasts *C. albicans*, *C. glabrata*, and *S. cerevisiae* were exposed to 50 µM of Hst-5 for 30 minutes and examined using fluorescent microscopy with a fixed exposure time (9 ms). Interestingly, in *C. glabrata* cells, Hst-5 was predominantly localized to the vacuole, whereas in *S. cerevisiae*, the peptide was found bound to the cell surface (Figure 4B). Thus, the resistance of *C. glabrata* and *S. cerevisiae* to Hst-5 may be due to the lack of cytoplasmic translocation of the antifungal peptide. To determine if the lack of Hst-5 uptake was due to a difference in cell surface binding, we exposed Hst-5 to the yeasts in the presence of sodium azide, which has been shown to prevent the internalization of Hst-5 [17]. Hst-5 uniformly bound to the cell surface of *C. albicans* and *S. cerevisiae* (Figure 4B). Therefore, the lack of cytoplasmic translocation of Hst-5 in *S. cerevisiae* was not due to the lack of cell surface binding. The uniform Hst-5 localization was not observed in *C. glabrata*. Instead, punctate regions were detected, which may correspond to ligand-receptor interactions and receptor-mediated endocytosis. Our data suggest that the resistance of *C. glabrata* and *S. cerevisiae* to Hst-5 is dependent on its ability to hinder the mechanism of cytoplasmic translocation.

### Histatin-5 induces perturbation at a spatially restricted site on the cell surface, leading to a rapid translocation of the peptide into the cytoplasm

**Figure 5:**
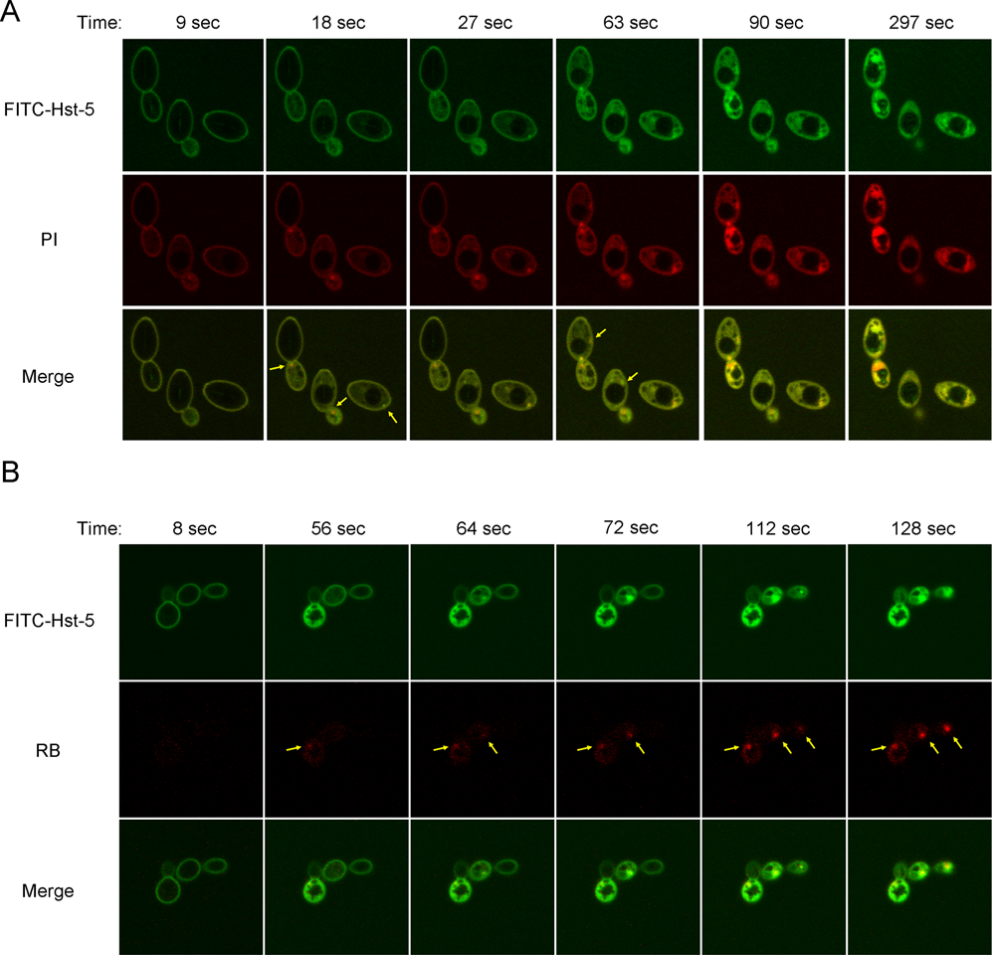
Hst-5 causes a single perturbation on the cell surface of *C. albicans*. (A) 50 µM FITC-Hst-5 was added to the buffer containing PI, and uptake of fluorescence was followed by time-lapse confocal microscopy at room temperature with frames recorded every 9 seconds for 7 minutes and 30 seconds. Six frames recorded at 9, 18, 27, 63, 90, and 297 seconds are shown. (B) 50 µM FITC-Hst-5 was added to the buffer containing RB, and uptake of fluorescence was followed by time-lapse confocal microscopy at room temperature with frames recorded every 8 seconds for a total of 6 minutes and 40 seconds. Six frames recorded at 8, 56, 64, 72, 112, and 128 seconds are shown.

Our previous experiments indicated that the cytoplasmic localization of Hst-5 was achieved in as little as 10 minutes after cells were exposed to the peptide. To further evaluate the internalization event, we used time-lapse confocal microscopy to visualize the process of Hst-5 uptake. 50 µM FITC-Hst-5 was added to *C. albicans* cells in a thin glass-bottom dish. Image acquisition started prior to the addition of the peptide, and frames were recorded every 9 seconds for a total of 7 minutes and 30 seconds. Hst-5 bound uniformly to the cell surface of the yeast almost immediately after addition of the peptide. Surprisingly, shortly afterwards, a green punctuate site was observed mostly on or near the plasma membrane in many cells (Figure 5A). Only one restricted region of concentrated Hst-5 was seen for each cell. Hst-5 rapidly spread throughout the cytoplasm by diffusion, resulting in a uniform accumulation of Hst-5 in the yeast cell (Figure 5A and Video S1). Concurrent with the internalization of Hst-5, we observed a rapid expansion of the vacuole with a parallel loss in cell volume in less than sixty seconds (Video S2). Eventually, at about 10 minutes, the vacuole collapsed in some cells (Video S2). The disruption in cellular membrane compartments is in agreement with the published electron microscope images of Hst-5 treated cells [43].

The observation of Hst-5 concentrated in a spatially constricted region is intriguing. One possible explanation is that Hst-5 caused a break on the plasma membrane and the peptide entered into the cytoplasm through the damaged site. If so, the site should also allow the entrance of other molecules. It is known that Hst-5 treatment of cells causes internalization of the cationic molecule propidium iodide (PI). Therefore, we asked whether PI enters cells from the same site as the antifungal peptide. Interestingly, PI was found localized as a single small dot either at or right under the cell surface that also colocalized with Hst-5 (Figure 5A). PI rapidly spread throughout the cytoplasm as was seen with FITC-Hst-5 (Figure 5A and Video S3). The co-localization of PI and Hst-5 at the same spatially restricted site suggests the existence of a breach of the cell surface by Hst-5. No internalization of PI was observed in the absence of Hst-5 (data not shown). Whereas, PI localized to a spatially restricted site on the cell surface when cells were treated with unconjugated Hst-5, confirming that FITC-Hst-5 and native Hst-5 have the same property in membrane perturbation (Figure S1). Among cells in several time-lapse experiments, we never observed a cell with more than one site of PI internalization. This was further supported by the acquisition of a three-dimensional image of the initial uptake of PI into the cytoplasm shortly after *C. albicans* was treated with FITC-Hst-5 (Video S4). Due to the rapid uptake of the fluorochrome, wide-field microscopy and constrained iterative deconvolution were used in place of confocal microscopy. The initial Hst-5 uptake site on the cell surface could not be observed in this image due to the high fluorescein signal on cell surface. Even *myo5* mutant cells, which have a lowered Hst-5 concentration threshold for cytoplasmic translocation, had solitary breaches on their cell surface (data not shown).

To further validate the findings that translocation of Hst-5 into the cytoplasm is mediated by the formation of a spatially restricted site on the cell surface of the yeast, *C. albicans* was treated with 50 µM FITC-Hst-5 in the presence of 5 µg/ml of rhodamine B (RB). The fluorchrome RB is useful indicator of membrane integrity and viability and is advantageous in this particular assay since it does not bind mitochondria or DNA [44],[45]. As observed with PI, the uncharged fluorochrome RB was internalized from a single regional area that colocalized with FITC-Hst5 (Figure 5B). Taken together the data indicates that Hst-5 induces a spatially localized breach on the cell surface of the *C. albicans*, leading to a disruption in its membrane integrity and a loss in viability.

**Figure 6:**
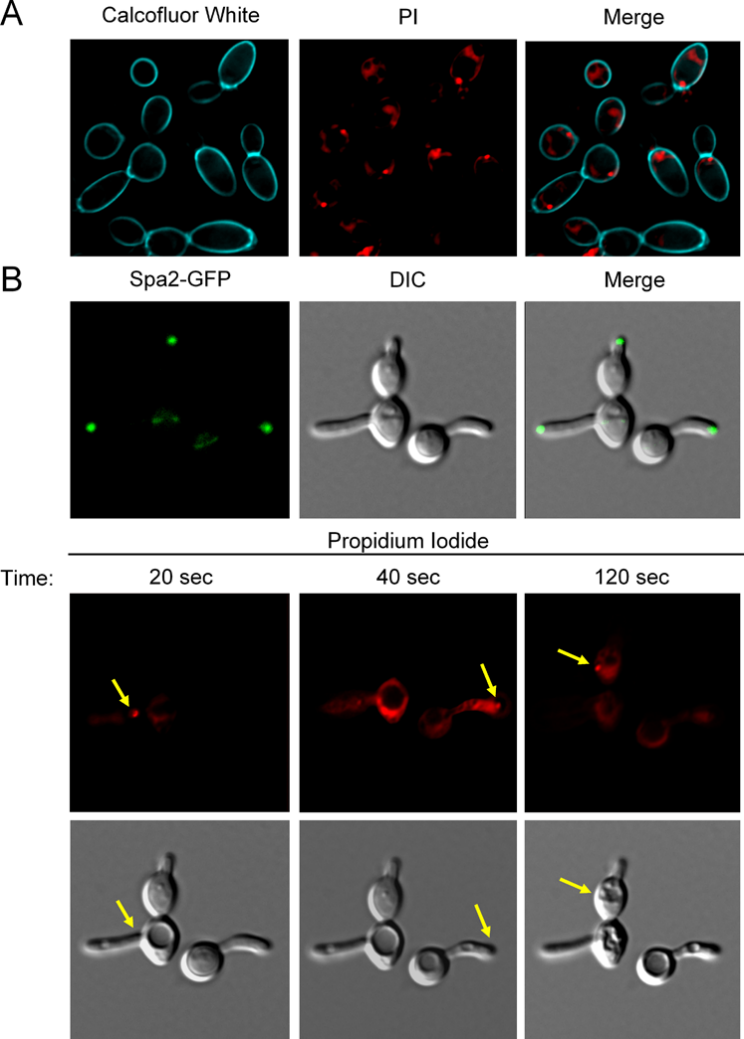
The Hst-5 induced perturbation of the membrane does not co-localize with a specific extracellular region. (A) 50 µM biotin-Hst-5 was added to *C. albicans* in buffer containing PI and calcofluor white. Uptake of PI was followed by time-lapse fluorescence microscopy at room temperature with frames recorded every 25 seconds for 10 minutes. The frame recorded at 400 seconds is shown. (B) *C. albicans* was incubated in water with 2% fetal calf serum and PI for 50 minutes at 37°C to induce Spa2-GFP to the site of polarized growth. Buffer containing 50 µM biotin-Hst-5 was added to the cells and uptake of PI was followed by time-lapse fluorescence microscopy at 37°C with frames recorded every twenty seconds. Frames recorded at 0, 20, 40, and 120 seconds are shown. The yellow arrows indicate the formation of a single breach site on the yeast.

Having shown that Hst-5 causes spatially restricted sites on the cell surface, we wanted to determine whether the site correlated with known cell surface markers. To evaluate whether the breach site was in regions where daughter cells had budded off, *C. albicans* cells were treated first with calcofluor white, and then with PI and biotin-conjugated Hst-5. The site of PI uptake did not correlate with bud scars (Figure 6A). This is consistent with the previous finding that Hst-5 is capable of killing spheroplasts of *C. albicans* [46]. We then examined whether there was any correlation between the site of the breach and cell polarity, which is linked to the active sites of secretion and endocytosis. Sterol and sphingolipid-rich raft domains are also concentrated at the site of active growth [47]. We performed time-lapse experiments with 50 µM biotin-Hst-5 and 5 µg/ml PI with *C. albicans* cells carrying GFP-tagged Spa2p, a component of the polarisome that controls cell polarity [48]. To facilitate analysis, hyphal cells with highly polarized growth were used. Spa2p was found to be localized to sites of polarized growth, such as the small bud, the bud neck, and the hyphal tip. However, the site of PI uptake did not correlate with the cellular localization of GFP-Spa2p (Figure 6B). Therefore, the spatially restricted site on the cell surface does not correlate with either of the two tested cell surface landmarks and is either a random manifestation on the cell surface or the uptake of the peptide is dependent on an unknown cellular marker found in association with either the cell wall and/or the plasma membrane of *C. albicans*.

## Discussion

The general mode of action of most α-helical cationic antimicrobial peptides is initiated by their net positive charge that attracts them to an anionic microbial surface. Following this electrostatic binding, the α-helical peptide is inserted into the plasma membrane, resulting in the release of cellular contents and/or lysis of the cell. Previous Hst-5 research has indicated that the mode of Hst-5 killing does not behave in this classical manner. Rather it has been suggested that Hst-5 binds the heat shock protein 70 (Ssa1/2) located on the cell wall and is subsequently transported across to the cytoplasm in a nonlytic manner [12],[20],[49]. Intracellular targeting by cytoplasmic Hst-5 to the mitochondria and the plasma membrane leads to cell death [6],[10],[49],[50]. Here we show for the first time that the uptake of Hst-5 is actually a dichotomous event. Under low physiological concentrations the peptide is internalized to the vacuole via receptor-mediated endocytosis. Whereas under moderate to high physiological conditions, uptake of Hst-5 to the cytoplasm is initiated by a direct translocation through a spatially restricted site on the plasma membrane, causing the initial fungicidal activity to the yeast by damage to the cell membrane. Uptake of Hst-5 is both a dynamic and stochastic process within the *Candida* population at low to moderate concentrations. The rapid uptake of Hst-5 into the cytoplasm and its subsequent killing of *C. albicans* most likely prevents the slower process of receptor-mediated endocytosis from happening in the same cells. On the other hand, when Hst-5 is below the critical concentration necessary to induce cytoplasmic translocation, the endocytic removal of Hst-5 from the cell surface likely lowers the build-up of Hst-5 on the cell surface, and therefore impedes the candidacidal activity of the antimicrobial peptide. These two opposing internalization pathways give rise to two cell populations with distinct Hst-5 localizations and outcomes.

Net cationic charge of Hst-5 plays a greater role than just the initial binding affinity to the surface of *C. albicans*. A single lysine substitution for a histidine or amidation of the C-terminus of Hst-5 analogs increases the candidacidal activity of the peptide by almost two-fold [35],[36]. Whereas, reduction of the net positive charge of Hst-5 by either amino acid substitution or an increase in pH markedly reduced the cytoplasmic translocation of the antimicrobial peptide without affecting binding to the cell surface. In support of this idea Dathe *et al.* showed that increasing the charge of a magainin 2 analog from +3 to +5 increased its antibacterial activity [51]. It has been postulated that mechanism of translocation of cationic peptides is based on the strong trans-negative membrane potential common to many pathogenic organisms. It is thought that the electrochemical gradient helps orient the cationic peptide to the membrane so that it may gain entrance to the polar membrane core and/or translocate across the exoplasmic to the cytoplasmic membrane leaflet [34]. The permeabilization of cellular membranes by magainin and platelet microbicidal proteins (PMPs) has been shown to require acidic phospholipids and a large membrane potential [52],[53]. Furthermore, while not affecting the initial binding event of Hst-5, *C. albicans* cells treated with uncouplers of the electrochemical gradient, such as carbonylcyanide-m-chlorophenylhydrazone, dinitrophenol, and sodium azide, as well as petite respiration-deficient mutants have increased resistance to Hst-5 [12],[18]. The membrane potential of *S. cerevisiae* has been measured at −71 mV, a value closer to that of mammalian cells (−90 to −110 mV), whereas *C. albicans* membrane potential is −120 mV, a value more in line with bacterial pathogens (−130 to −150 mV) [34],[54],[55]. The trans-negative electrochemical gradient may potentially act as an underlying mechanism providing Hst-5 resistance to *S. cerevisiae* and Hst-5 susceptibility to *C. albicans*. This mechanism could provide species-specific targeting of the peptide while preserving nonpathogenic microbial communities and host tissue.

One of the most intriguing aspects of this study was the demonstration that the primary event in the inevitable killing of *C. albicans* was due to the concentration dependent formation of a single disruption site on the plasma membrane, visualized by the rapid influx of the fluorochromes PI and RB and Hst-5 through the disruption on the cell surface of *C. albicans*. The existence of a breach on the plasma membrane is also evident from the rapid expansion of the vacuole and the loss of cell volume. In *S. cerevisiae*, the vacuole expands when cells are shifted to a hyposmotic condition [56]. The rapid expansion of the vacuole in Hst-5 treated cells could be a response to the loss of ions and other small solutes from the cytoplasm. The disruption site is likely responsible for the rapid efflux of ATP and K+ that are linked to the killing of *C. albicans*. The size of the holes should be small, as only proteins smaller than 4 kDa were found to be released into the culture [28]. To our knowledge only one other antimicrobial peptide, the amphibian skin peptide dermaseptin s3, has been observed to induce a single breach site on the surface of a microorganism [57]. The dependence of Hst-5 internalization on the membrane potential may provide an explanation for a single rupture per cell, as once there is one site of perturbation, the membrane potential is lost, and therefore, prevents a second rupture. The dependence on the existence of the membrane potential may explain why Hst-5 had little lytic effect on vesicles *in vitro* [9]. Moreover, Hst-5 is capable of targeting and disrupting the electrochemical gradient of isolated mammalian mitochondria, as well as the mitochondria of *C. albicans* and *Leishmania* species [6],[58],[59]. The site of the rupture seems to be random, as it does not correlate to regions where daughter cells bud off or to the region of polarized growth. The randomness of the solitary disruption site further indicates that Hst-5 may act on the plasma membrane directly, but not through a particular cellular landmark. However, at this time we cannot definitively exclude the possibility that the formation of the Hst-5 spatially restricted site is due to its greater affinity to an unknown cellular marker on the surface of *C. albicans* rather than the strong electrochemical gradient of the plasma membrane.

Nonetheless, two additional lines of evidence suggest that the Hst-5 interaction with the plasma membrane initiates a mode of action that weakens the stability of membrane bilayer. First, our research indicates that the deletion of the class I myosin and the depolymerization of F-actin by CytoA and LatA markedly reduces the threshold necessary for the cytoplasmic translocation of Hst-5. The cortical actin cytoskeleton is important for the stability of the plasma membrane under hyperosmotic conditions, as *S. cerevisiae act1* mutants and *C. albicans myo5* mutants are extremely sensitive to salt stress [33],[60]. Furthermore, the high-osmolarity glycerol pathway (Hog1p), involved in adaptation to osmotic and oxidative stress [61]–[63], is required to prevent severe growth defects in the *C. albicans myo5* mutants [60]. Second, Hst-5 is shown to activate the Hog1 pathway in *C. albicans* [15]. In *S. cerevisiae*, the Hog1 pathway is activated by a reduction of turgor pressure in response to hyperosmotic stress that induces water efflux or by treatment with nystatin, a membrane-permeabilizing antifungal drug that causes leakage of low molecular weight cytosolic components [64]. Moreover, Hst-5 shows strong synergistic killing of *C. albicans* when combined with amphotericin B [65],[66]. The synthetic interaction between Hst-5 and amphotericin B and the fact that both activate the Hog1 pathway suggests that Hst-5 and polyene macrolides (eg. amphotericin B and nystatin) have overlapping functions.

In summary, Hst-5 is not a classical channel forming cationic peptide. However, we do show in this report that Hst-5 induces perturbation at a spatially restricted site on the plasma membrane. Unlike classical channel forming antimicrobial peptides, this action requires an energized membrane and causes disruption at one region of the plasma membrane. This mechanism of cell membrane disruption may provide species-specific killing with minimal damage to microflora and the host, and may be used by many other weakly amphipathic antimicrobial peptides.

## Materials and Methods

### Yeast strains and growth conditions

The following fungal strains were used in this study: *C. albicans* SC5314 (wild-type clinical isolate); *C. albicans vps36* (BWP17 *Cavps36*Δ::*UAU1*/*Cavps36*Δ::*URA3*) and *C. albicans snf7* (BWP17 *Casnf7*Δ::*UAU1*/*Casnf7*Δ::*URA3*) (gifts from A. Mitchell, [67]); *C. albicans COU46* (CAI4 *Camyo5*::*hisG*/*Camyo5*::*hisG*) (gift from M. Whiteway, [68]); *C. albicans SPA2-GFP* (BWP17 *SPA2/SPA2-GFP-URA3*); *C. glabrata* BG2 (gift from B. Cormack, wild-type clinical isolate, [69]); and *S. cerevisiae* BY4741 (*MATa*, *leu2*Δ*0*, *met15*Δ*0*, and *ura3*Δ*0*). All of the yeast strains were maintained on YPD plates [1% (w/v) yeast extract, 2% (w/v) peptone, and 2% (w/v) glucose]. Prior to Hst-5 localization assays, the cells were grown overnight at 30°C in 5 ml YPD broth. A 1/50 dilution of the overnight culture was suspended in fresh 5 ml YPD and grown for an additional 4 hours at 30°C to obtain a mid-log phase culture at which time the optical density was determined (OD_595_ of 1.0 = 3×10^7^ cells/ml) using a Beckman Coulter DU 800 spectrophotometer to obtain a cell population of 10^6^ cells/ml.

### Peptides

Unconjugated Hst-5 and FITC- and biotin-labeled Hst-5 (DSHAKRHHGYKRKFHEKHHSHRGY) and FITC-labeled Hst-5 m68 (DSHAKRHHGYKR**E**FHEKHHSH**G**GY) were synthesized and purified by Genemed Synthesis, Inc. (San Francisco, CA). The identity and purity of the peptides were confirmed by mass spectrometry. Both FITC-Hst-5 and biotin-Hst-5 have been shown to have similar levels of candicidal activity when compared against unlabeled Hst-5 [10],[13],[49].

### Hst-5 localization studies

The intracellular localization of FITC-Hst-5 (5, 10, 20, and 50 µM) and FITC-m68 (5, 10, 20, 50, and 200 µM) was investigated either alone or in a double-labeling experiment using FM4-64 (Molecular Probes, Inc. Eugene, OR). Yeast cells in 50 µl (∼10^6^ cells/ml) were incubated for 30 minutes at 30°C with 10 µM FITC-Hst-5 and 10 µM FM4-64; the cells were then washed twice with 10 mM NaN_3_ and 10 mM NaF in 20 mM PBS buffer, and analyzed immediately by wide-field fluorescence microscopy.

To depolarize F-actin, cells were treated with either 5 µM cytochalasin A or 50 µM latrunculin A (Sigma, St. Louis, MO) for 1 hour at room temperature. The control cells (wild-type and *myo5*) were treated with the equivalent volume of the DMSO solvent (0.5%). The cells were then exposed to 5 µM of FITC-Hst-5 for 30 minutes at 30°C. The cells were then washed twice with buffer containing NaN_3_ and NaF and analyzed by wide-field fluorescence microscopy and flow cytometry.

For live cell imaging, 300 µl of a 100 µg/ml solution of concanavalin A (MP Biomedicals, LLC. Solon, Ohio) was coated onto a sterile 0.17 mm glass bottom dish (WillCo Wells BV, Amsterdam, Denmark). The wells were incubated for 1 hour at room temperature and then washed three times with water. A 300 µl buffer suspension of ∼2×10^6^ *C. albicans* cells were aliquoted onto the well and incubated at room temperature. After settling and binding for 15 minutes unbound cells were washed away [70]. Buffer containing 50 µM of either unconjugated Hst-5 or FITC-Hst-5 and 5 µg/ml PI or RB was added to the cells and uptake of fluorescence was followed by time-lapse confocal microscopy. To determine the position of site specific breach by Hst-5 in relation to known cellular markers, cells were either observed using 2 µg/ml calcofluor white (Sigma, St. Louis, MO) and 5 µg/ml PI in PBS buffer or the *C. albicans* Spa2-GFP strain was grown in the presence of PI and 2% fetal calf serum in water. Buffer containing 50 µM biotin-Hst-5 was added to the cells and uptake of PI was followed by time-lapse wide-field fluorescence microscopy at room temperature and 37°C, respectively.

### Fluorescence microscopy

Wide-field fluorescence images were obtained on either Zeiss Axioplan 2 or the inverted Zeiss Axio Observer.Z1 Microscope (Carl Zeiss MicroImaging, Inc. Thornwood, NY) fluorescent system, equipped with the AttoArc HBO 100 and the X-Cite series 120 mercury lamps, respectively. Images were taken using a 100× NA 1.4 objective. Both fluorescence microscopes were equipped with GFP, RFP, and DAPI filter sets.

Data sets were obtained as 10–20 optical sections per wavelength spaced 0.2 µm apart along the Z-axis. Out of focus information was removed using a constrained iterative deconvolution algorithm. During the experiment cells were kept at either a constant 30° or 37°C using the TempModule S system on the microscope. Processing was done on a PC using the software packages Axiovision 3.1 and 4.6.3, as well as Photoshop (Adobe Systems Inc., Mountain View, CA).

Confocal laser scanning microscopy was performed on an inverted LSM510 laser scanning microscope (Carl Zeiss, Göttingen, Germany) using a Plan-Apo 100×/1.4 NA lens. For the simultaneous detection of fluorescein-labeled peptides and the fluorochromes PI or RB, the 488-nm line of the argon ion laser and the light of a 543-nm helium neon laser were directed over an HFT UV/488/543/633 beam splitter, and the fluorescence was detected using an NFT 545 beam splitter in combination with a BP 500–550 band pass filter for fluorescein detection and an BP 565–615 band pass filter for PI and RB detection.

### FACS analysis

The distribution of FITC-labeled Hst-5 over the cell population was investigated by using a dual laser fluorescence-activated cell sorter (BD FACSCalibur System, Becton Dickinson, San Jose, CA). The results were analyzed with the software package CellQuest Pro (version 5.1.1) provided by Beckton Dickinson.

### Flow cytometric cell sorting and candidacidal activity of histatin-5

*C. albicans* cells were incubated for 30 minutes with 10 µM of FITC-Hst-5 at 30°C. The cells were then washed twice with 20 mM PBS buffer and under went flow cytometric cell sorting using the DAKO Cytomation MoFlo Flow Cytometer (DAKO, Glostrup, Denmark). The results were analyzed on a PC using the software package Summit (version 4.0) provided by DAKO. The cells were sorted by gating the two peaks of the histogram representing vacuolar and cytoplasmic localization of Hst-5. Cellular localization of Hst-5 was confirmed with fluorescence microscopy. The sorted cells were then plated onto YPD plates and incubated overnight at 30°C (data as a mean±1SD of triplicate cultures).

### Determination of charge for Hst-5 and m68

Determination of charge for Hst-5 and m68 were done using PROTEIN CALCULATOR v3.3 (www.scripps.edu/∼cdputnam/protcalc2.html). The pKa values for the individual amino acids are from Stryer Biochemistry, 3^rd^ edition. The software was designed by Chris Putnam at the Scripps Research Institute cdputnam@scripps.edu.

## Supporting Information

Figure S1 Unlabeled Hst-5 induced internalization of the fluorchrome propidium iodide from spatially restricted sites on the cell surface. 50 µM Hst-5 (unconjugated) was added to the buffer containing PI, and uptake of fluorescence was followed by time-lapse confocal microscopy at room temperature, with frames recorded every 9 seconds for 7 minutes and 30 seconds. Six frames recorded at 9, 27, 45, 90, and 297 seconds are shown.
(2.00 MB TIF)

Video S1 Cytoplasmic translocation of FITC-Hst-5 into the yeast *C. albicans*. The uptake of fluorescence after the addition of 50 µM FITC-Hst-5 to *C. albicans* cells. The images were recorded by time-lapse confocal microscopy at room temperature with frames recorded every 9 seconds for a total of 7 minutes and 30 seconds.
(6.42 MB MOV)

Video S2 Rapid vacuole expansion and deformation of *C. albicans* after the addition of Hst-5. The morphological change to the yeast *C. albicans* after the addition of 50 µM Hst-5. The images were recorded by time-lapse microscopy at room temperature with frames recorded every 15 seconds for a total of 15 minutes.
(2.20 MB MOV)

Video S3 Cytoplasmic translocation of propidium iodide into the yeast *C. albicans*. The uptake of PI after the addition of 50 µM FITC-Hst-5 to *C. albicans* cells. The images were recorded by time-lapse confocal microscopy at room temperature with frames recorded every 9 seconds for a total of 7 minutes and 30 seconds.
(6.42 MB MOV)

Video S4 Localization of FITC-Hst-5 and propidium iodide using three-dimensional imaging. Buffer containing 50 µM FITC-Hst-5 and 5 µg/ml PI was added to the cells, and uptake of fluorescence was followed by wide-field time-lapse microscopy. The three-dimensional image was acquired from deconvolution using fifteen 0.2 micron optical sections.
(2.10 MB MOV)
