# Supplementary figures and images for: The Antimicrobial Peptide Histatin-5 Causes a Spatially Restricted Disruption on the Candida albicans Surface, Allowing Rapid Entry of the Peptide into the Cytoplasm
Source: PLoS Pathog. 2008 Oct 31;4(10):e1000190. doi: 10.1371/journal.ppat.1000190 (PMC2568956; doi:10.1371/journal.ppat.1000190)

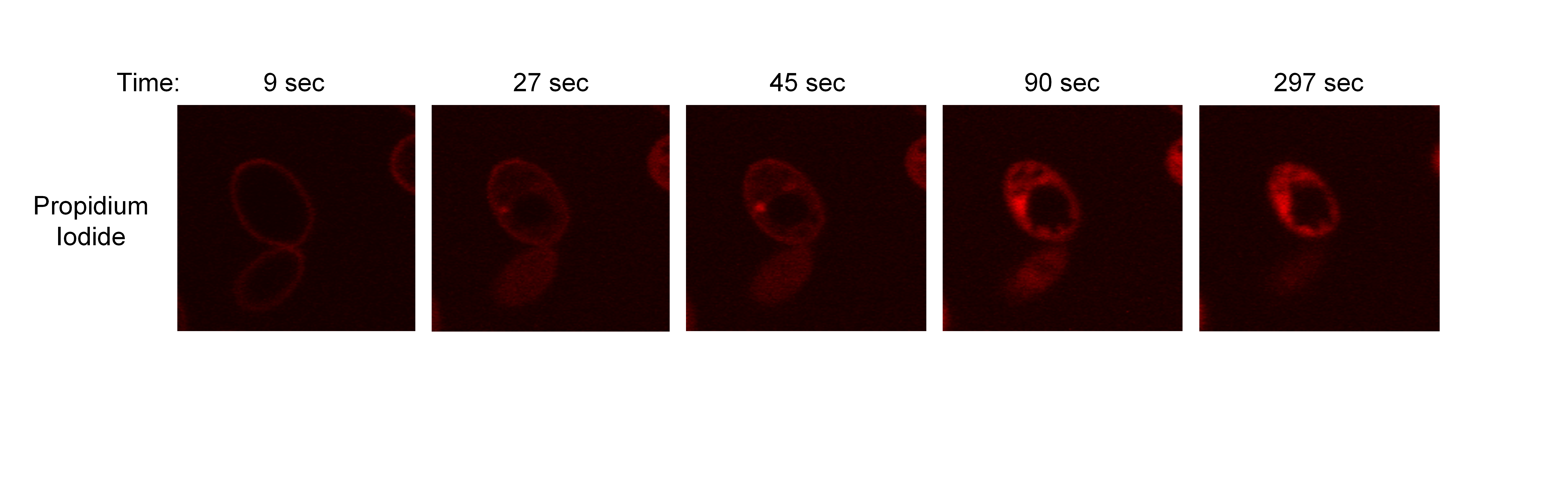

Supplement: Figure S1 — Unlabeled Hst-5 induced internalization of the fluorchrome propidium iodide from spatially restricted sites on the cell surface. 50 µM Hst-5 (unconjugated) was added to the buffer containing PI, and uptake of fluorescence was followed by time-lapse confocal microscopy at room temperature, with frames recorded every 9 seconds for 7 minutes and 30 seconds. Six frames recorded at 9, 27, 45, 90, and 297 seconds are shown. (2.00 MB TIF) [file ppat.1000190.s001.tif]
